# Levels and Age Dependency of Neurofilament Light and Glial Fibrillary Acidic Protein in Healthy Individuals and Their Relation to the Brain Parenchymal Fraction

**DOI:** 10.1371/journal.pone.0135886

**Published:** 2015-08-28

**Authors:** Mattias Vågberg, Niklas Norgren, Ann Dring, Thomas Lindqvist, Richard Birgander, Henrik Zetterberg, Anders Svenningsson

**Affiliations:** 1 Department of Pharmacology and Clinical Neuroscience, Section of Neuroscience, Umeå University, Umeå, Sweden; 2 UmanDiagnostics AB, Umeå, Sweden; 3 Department of Radiation Sciences, Section of Diagnostic Radiology, Umeå University, Umeå, Sweden; 4 Clinical Neurochemistry Laboratory, Institute of Neuroscience and Physiology, the Sahlgrenska Academy at the University of Gothenburg, Mölndal, Sweden; 5 UCL Institute of Neurology, Queen Square, London, United Kingdom; Medical University of Innsbruck, AUSTRIA

## Abstract

**Background:**

Neurofilament light (NFL) and Glial Fibrillary Acidic Protein (GFAP) are integral parts of the axonal and astrocytal cytoskeletons respectively and are released into the cerebrospinal fluid (CSF) in cases of cellular damage. In order to interpret the levels of these biomarkers in disease states, knowledge on normal levels in the healthy is required. Another biomarker for neurodegeneration is brain atrophy, commonly measured as brain parenchymal fraction (BPF) using magnetic resonance imaging (MRI). Potential correlations between levels of NFL, GFAP and BPF in healthy individuals have not been investigated.

**Objectives:**

To present levels of NFL and GFAP in healthy individuals stratified for age, and investigate the correlation between them as well as their correlation with BPF.

**Methods:**

The CSF was analysed in 53 healthy volunteers aged 21 to 70 (1 sample missing for GFAP analysis) and 48 of the volunteers underwent determination of BPF using MRI.

**Results:**

Mean (±SD) NFL was 355 ng/L (±214), mean GFAP was 421 ng/L (±129) and mean BPF was 0.867 (±0.035). All three biomarkers correlated with age. NFL also correlated with both GFAP and BPF. When controlled for age, only the correlation between NFL and GFAP retained statistical significance.

**Conclusions:**

This study presents data on age-stratified levels of NFL and GFAP in the CSF of healthy individuals. There is a correlation between levels of NFL and GFAP and both increase with age. A correlation between NFL and BPF was also found, but did not retain statistical significance if controlled for age.

## Introduction

Neurofilaments are important structural components in axons in the peripheral as well as the central nervous system (CNS). They compose a heteropolymer of the three subunits: neurofilament light (NFL), medium and heavy chains (NFH) [1]. Elevated levels of NFL can be measured in cerebrospinal fluid (CSF) following axonal damage in the CNS [2–4]. Both levels of NFH and NFL have been shown to correlate with age [5–9]. Glial fibrillary acidic protein (GFAP) is another structural component of astrocytes. Elevated GFAP levels in CSF can be seen in cases of astrocytic damage [4]. As with NFL, correlation between levels of GFAP in CSF and age has been described [10], but the relationships have not been thoroughly investigated for either marker in healthy individuals.

It has been shown that global brain atrophy occurs with normal aging in healthy individuals [11]. This can be measured as a decrease in brain parenchymal fraction (BPF), here defined as the total volume of brain parenchyma divided by the total intracranial volume. The BPF has been shown to correlate negatively with age in healthy individuals [12–14].

Both NFL and GFAP are potential clinical biomarkers of central nervous system (CNS) tissue damage, but in order to properly interpret individual clinical values the normal range in relation to the patients’ age needs to be known. It can also be hypothesised that an age-dependency of tissue damage markers such as NFL and GFAP could correlate with the age-dependency of BPF. The goal of this study was to investigate the relationship between levels of NFL and GFAP in CSF of healthy individuals, and their relationships to BPF.

## Methods

### Study population

Lumbar puncture was performed in 53 (27 females) neurologically healthy volunteers. The volunteers were interviewed by a research nurse to screen for any neurological disease or any first degree relatives with such diseases. Individuals with morbidity not directly related to the nervous system were accepted for inclusion. Median age was 33.7 years (interquartile range 21.3; range 21 to 69.9). A subset of 48 (24 female) of the individuals also volunteered for magnetic resonance imaging. The median age of the individuals who underwent MRI was 33.0 years (interquartile range 18.4; range 21.3 to 69.9 years).

### Analysis of NFL and GFAP

Level of NFL in CSF was measured using ELISA (UmanDiagnostics NF-light assay, UmanDiagnostics AB) according to the ELISA kit instructions. The manufacturer of the kit states the assay’s lower limit of quantification as 61 ng/l and mean intra- and inter-assay coefficient of variation as 4% and 6% respectively. Levels of NFL were analysed by two different labs, producing dual NFL values for each volunteer.

Level of GFAP in CSF was measured using an in house ELISA based on polyclonal antibodies as previously described in detail [15]. The assay has a lower limit of quantification of 70 ng/l and intra- and inter-assay coefficients of variation of 4% and 8%, respectively.

### Magnetic resonance imaging

The MRI was acquired using a 3T Philips scanner. Five echoes were acquired at multiples of 17.5 ms. Four saturation delays were acquired at 150, 570, 1550, and 3370 ms. This resulted in 20 axial images per slice at different echo times and saturation delays, allowing for quantification of the relaxation rates R1 and R2 and the proton density. The repetition time was 3.5 seconds for 30 slices of 4.5 mm slice thickness, with a gap of 0.5 mm. The field of view was 230 mm, and the in-plane resolution was 1 mm. The BPF was determined using the SyMap method [13], based on MRI relaxometery.

### Ethics statement

The study was approved by the Regional Ethical Review Board of Umeå University and all participants gave written informed consent.

### Statistics

**Fig 1:**
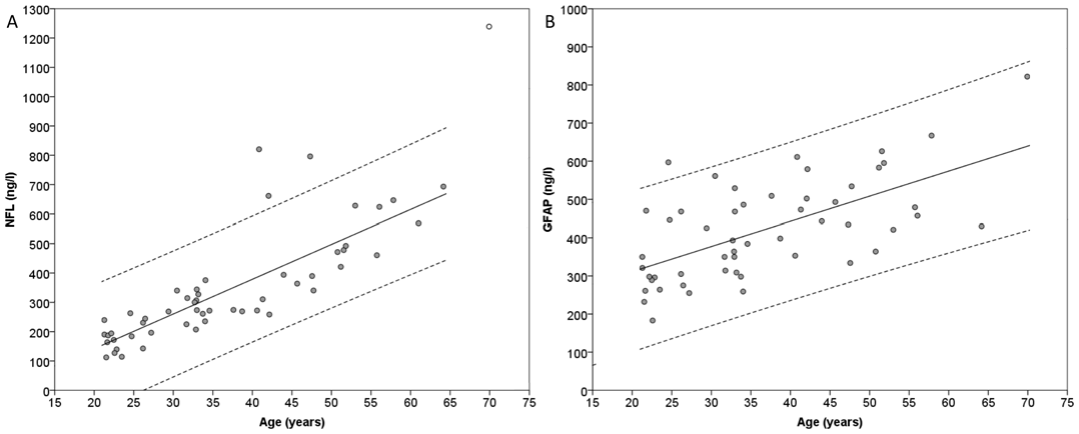
Levels of NFL and GFAP plotted against age. The levels of NFL (A) and GFAP (B) in CSF plotted against age. The dashed lines represent 95% prediction intervals. The linear regression between NFL and age had an R^2^-value of 0.649, a coefficient of 11.8 ng/l/year (SE = 1.23, p<0.001) and an intercept at -95 ng/l (p = 0.05). The linear regression between GFAP and age had an R^2^-value of 0.394, a coefficient of 6.56ng/l/year (SE = 1.15, p<0.001), and an intercept at 180 ng/l (p<0.001). One outlier case (circle filled with white) was excluded from the calculation of the regression line and prediction interval regarding the regression between NFL and age. NFL = Neurofilament Light. GFAP = Glial Fibrillary Acidic Protein.

All statistics were computed using SPSS (IBM Corp. 2013 SPSS Statistics for Windows, version 22.0). The Shapiro-Wilk test in conjunction with inspection of histograms and the skewness statistic were used to test for normality of distribution. Age, NFL and BPF were all non-normally distributed. Spearman’s rho was used for bivariate correlation testing. Partial rank correlations was used for non-parametric partial correlation testing. Linear regression was used to evaluate the relationship between age and NFL, GFAP and BPF. Wilcoxon signed rank test was used to test for inter-lab differences in NFL levels. An α-level of 0.05 was chosen for determination of statistical significance. The Bonferroni-Holm method was applied to control for familywise error rate in the significance testing of the partial correlations. One individual (specified in Fig 1) was excluded from correlation testing and regression line estimation regarding NFL on the basis of having an outlier NFL value and no other individuals of the same age was available for comparison.

## Results

### Comparison of NFL values between labs

The mean NFL values for the whole group (±SD) were 332ng/L (±221 ng/L) for lab one and 378 ng/L (±209 ng/L) for lab two. The difference was statistically significant (p<0.001). However, the individual values obtained from the two labs displayed a high degree of correlation (r = 0.977, p < 0.001). The mean value of NFL from the two labs was used for all calculations below.

### NFL and GFAP

One GFAP sample was missing (data lost), resulting in GFAP values for 52 individuals, NFL values for 53 individuals and BPF values for 48 individuals. All three biomarkers were available for 47 individuals. The mean values of NFL, GFAP and BPF are presented in Table 1. The values for NFL and GFAP are plotted in relation to age in Fig 1 and linear regressions with age as the independent variable are shown. Both NFL (r = 0.870) and GFAP (r = 0.595) correlated with age (p<0.001). Levels of NFL and GFAP also correlated with each other (r = 0.655, p<0.001).

**Table 1:** Mean values of NFL, GFAP and BPF.

Age (years)	N^NFL^ (median age; IQR)	Median NFL (IQR)	N^GFAP^ (median age; IQR)	Median GFAP(IQR)	N^BPF^ (median age; IQR)	Median BPF (IQR)
**<30**	17 (22.8; 4.46)	187 (94.0) ng/l	17 (22.8; 4.46)	298 (173) ng/l	17 (22.8; 4.46)	0.887 (0.0357)
**30 to <40**	15 (33.0; 1.37)	274 (66.5) ng/l	15 (33.0; 1.37)	384 (172) ng/l	14 (33.0; 1.72)	0.867 (0.0230)
**40 to <60**	18 (47.6; 9.99)	466 (275) ng/l	18 (47.6; 9.99)	486 (155) ng/l	14 (46.5; 9.41)	0.866 (0.0511)
**60+**	3 (64.2; 61.0–70.0*)	693 (568–1239*) ng/l	2 (67.0; 64.2–70.0*)	626 (430–822*) ng/l	3 (64.2; 61.0–70.0*)	0.794 (0.770–0.811)

The mean values of NFL, GFAP and BPF, stratified by age.

* denotes that the numbers indicate range instead of IQR

IQR = Interquartile range

NFL = Neurofilament Light

GFAP = Glial Fibrillary Acidic Protein

BPF = Brain Parenchymal Fraction.

### MRI

The MRI of each individual was examined by an experienced neuroradiologist in conjunction with an experienced neurologist. None of the 48 individuals who underwent MRI had any MRI findings that were determined to be clinically significant. However, ten of the individuals had abnormal findings of benign appearance assumed to have minimal impact on the measurements of NFL, GFAP and BPF. These included one case of Arnold Chiari malformation type 1, one case of hyperostosis, one case of sinusitis, two cases of pineal gland cysts, one case of cavernoma and four cases of unspecific white matter changes of gliotic appearance.

**Fig 2:**
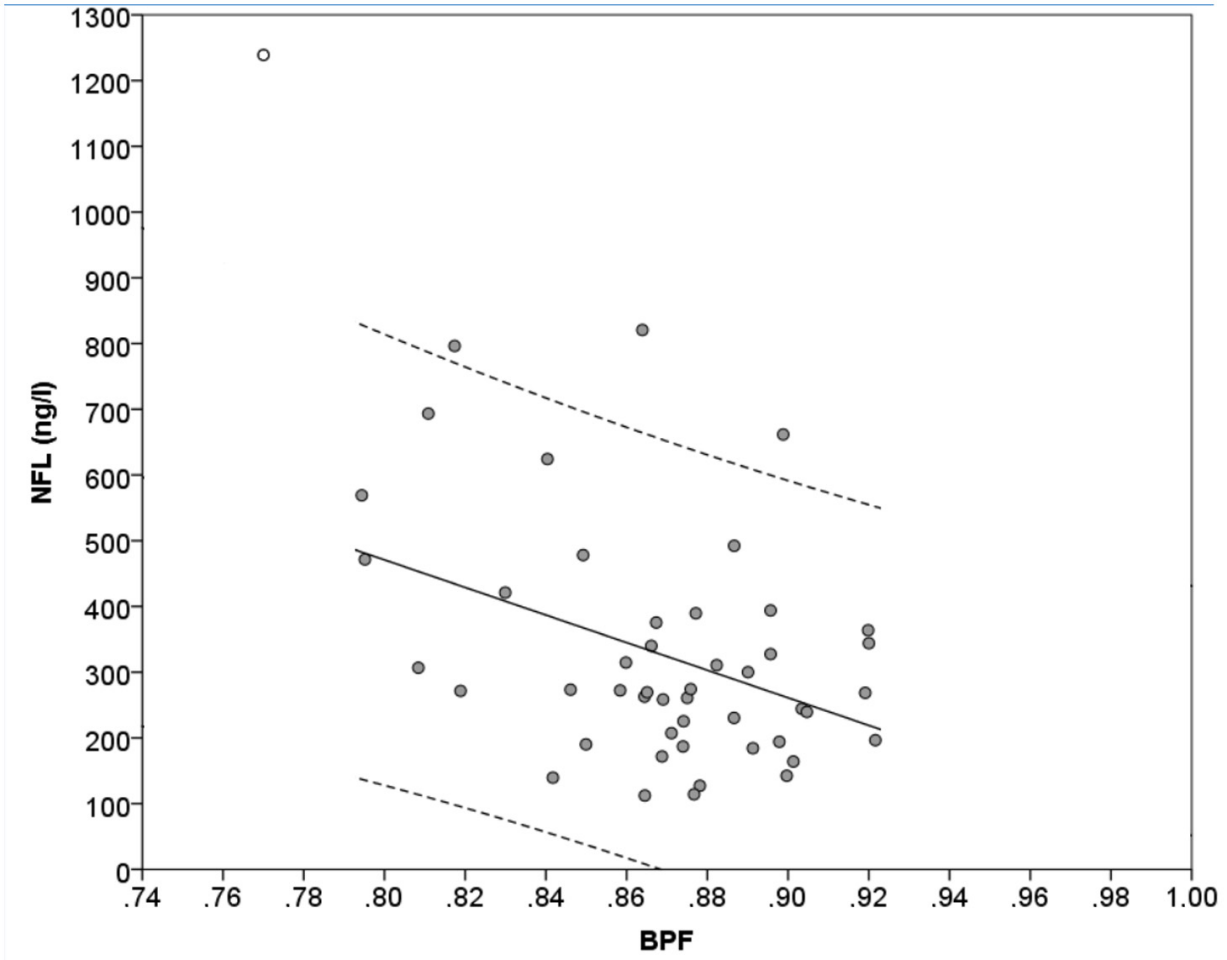
Level of NFL plotted against BPF. The level of NFL plotted against BPF. The dashed lines represent 95% prediction intervals. The linear regression had an R^2^-value of 0.154, a coefficient of -210ng/l per 0.1 change in BPF (SE = 73, p = 0.006) and an intercept at 2150 ng/l (p = 0.002). One outlier case (circle filled with white) was excluded from the calculation of the regression line and prediction interval regarding the regression between NFL and BPF. NFL = Neurofilament Light. BPF = Brain Parenchymal Fraction.

The BPF values are presented in Table 1. The BPF correlated negatively with levels of NFL (r = -0.308, p = 0.035) and with age (r = -0.396, p = 0.005) but not with GFAP (p = 0.151). Values of NFL are presented plotted against values of BPF in Fig 2.

### Partial correlation

Partial correlations while controlling for age are shown in Table 2. In this analysis only the correlation between NFL and GFAP remained statistically significant.

**Table 2:** Partial correlations while controlling for age.

Partial correlations while controlling for age			
	NFL	GFAP	BPF
NFL	-	0.38 (p = 0.027) *	-0.001 (p = 1.0)
GFAP	-	-	-0.003 (p = 1.0)

Partial rank correlations between the values of NFL, GFAP and BPF while controlling for age. The p-values are adjusted using Bonferroni-Holm correction. One outlier case (specified in Fig 1) was excluded from correlation testing involving NFL. This exclusion did not affect any statistical significances.

* denotes statistically significant correlation

NFL = Neurofilament Light

GFAP = Glial Fibrillary Acidic Protein

BPF = Brain Parenchymal Fraction.

## Discussion

This study presents age-stratified levels of NFL and GFAP, showing an increase in both biomarkers with increasing age. Both biomarkers also correlated with each other and NFL, but not GFAP, correlated with BPF. When correlations were controlled for age using partial correlations, the correlation between NFL and GFAP decreased but retained statistical significance. This correlation may indicate an individual variation in axonal and glial cell turnover that is separate from the effect of normal ageing. Future studies investigating longitudinal data from the same individuals could be helpful in clarifying this. The correlation between NFL and BPF was markedly reduced and did not retain statistical significance when controlled for age, indicating that the correlation between the variables in a cross-sectional setting entirely or to a great degree is based on their respective association to age. It is interesting to note that previous data on the correlation between NFL and age show that while it can be seen in the healthy controls it is absent in patients with the MS associated entity clinically isolated syndrome, perhaps indicating that the pathological neurodegeneration caused by the disease is of such a magnitude that it masks the NFL levels associated with the natural aging process [6,9]

Even if the three biomarkers ultimately would represent the same age-related degenerative process a lack of correlation could perhaps be explained by the biological difference in dynamics between them. A decrease in BPF is commonly interpreted as representing an accumulation of tissue damage over time with only a smaller component of dynamic variation due to physiological factors such as hydration status. The levels of NFL and GFAP, on the other hand, are more dynamic and quickly elevated following pathologic tissue damage [16]. The dynamics of the normal level of NFL and GFAP are not well known. It is furthermore possible that an individual’s current levels of NFL and GFAP could represent current tissue damage resulting in subsequent, but not concurrent, decrease in BPF. This hypothesis is strengthened by the fact that one previous study looking at correlation between NFL and normalised brain volume (NBV, a measurement similar to BPF) saw no correlation in cross-sectional testing but presented a correlation between level of NFL and subsequent decrease in NBV [9]. Further longitudinal studies comparing levels of CSF biomarkers with subsequent change in BPF could increase our understanding of age-related neurodegeneration further.

The reason for age dependency is not thoroughly understood regarding any of the three biomarkers investigated in this study. The correlations with age suggest ongoing CNS tissue damage with normal ageing but the biological cause of this is not well known. It could perhaps be hypothesised that subclinical cerebrovascular changes are a factor, since cerebrovascular pathology is common, increases with higher age and has previously been associated with both lower BPF [17] and higher levels of NFL and GFAP in CSF [16]. Further research is needed to elucidate this matter.

It is important to note that 53 individuals are not enough to unequivocally define the ranges of normal variation, especially for older age groups where the number of volunteers was low. However, given the difficulty in recruiting healthy volunteers to provide CSF samples due to many perceiving the procedure of lumbar puncture to be uncomfortable, this moderately sized sample set provides an important starting point for assessing the normal ranges of these biomarkers.

This study presents normal values of NFL, GFAP and BPF stratified for age. Knowledge of the normal values for healthy individuals is important to be able to properly interpret individual values in the setting of clinical diagnostic or prognostic considerations. All three biomarkers have the potential to give clinically meaningful information, for example in the case of multiple sclerosis where NFL and GFAP have been shown to correlate with disease activity and progression, respectively. [10] Furthermore, change in BPF has been shown to correlate with future development of disability in MS patients [18]. There was a statistically significant difference between the NFL values from the two labs, indicating that there might be a need to confirm the normal values of NFL locally to be able to correctly interpret values on the boundary of normality. However, when considering the potential use of NFL as a clinical biomarker, the magnitude of the difference was in a range that most likely is of minor clinical significance. The values of GFAP were only analysed in one lab and inter-lab difference could therefore not be studied.
